# Comparative effectiveness and safety of intravenous methylprednisolone and tacrolimus monotherapy in ocular myasthenia gravis with unsatisfactory prednisone responses: a retrospective study

**DOI:** 10.1186/s13023-024-03025-z

**Published:** 2024-01-19

**Authors:** Kai-Yue Zhang, Wei-Wei Duan, Yue-Bei Luo, Yi Li, Jue Hu, Huan Yang

**Affiliations:** 1 Department of Neurology, Xiangya Hospital, Central South University, Xiangya Road, Kaifu District, Changsha, 410008 China; 2 Department of Neurology, Changsha Central Hospital, Changsha, China

**Keywords:** Ocular myasthenia gravis, Intravenous methylprednisolone, Tacrolimus, Monotherapy, Clinical effectiveness, Initial exacerbation

## Abstract

**Background:**

Oral prednisone has been recognized as the first-line therapy for the treatment of ocular myasthenia gravis (OMG). However, its long-term use is complicated by numerous adverse effects and is ineffective for some OMG patients in reaching remission. This study aimed to evaluate the effectiveness and safety of intravenous methylprednisolone (IVMP) and tacrolimus monotherapy for OMG patients with unsatisfactory responses to conventional prednisone therapy.

**Methods:**

We retrospectively reviewed 57 OMG patients who had not achieved satisfactory improvement after prednisone therapy and thereby received IVMP or tacrolimus monotherapy for at least 6 months. Ocular symptoms were evaluated by the ocular-quantitative MG (QMG) score at each time point. A ≥ 2-point fall in ocular QMG score was defined as the cut-off point to indicate clinical improvement. Logistic regression analysis was performed to identify factors associated with the efficacy of IVMP at discharge. Adverse events were recorded.

**Results:**

Both IVMP and tacrolimus monotherapy demonstrated significant clinical efficacy, with no statistical differences observed at the study endpoint. The proportions of patients who reached the cut-off point for efficacy evaluation were higher in the IVMP group than in the tacrolimus group (1, 3, and 6 months: 51.7% (15/29) vs 12.0% (3/25), *p* = 0.002; 69.0% (20/29) vs 40.0% (10/25), *p* = 0.033; 69.0% (20/29) vs 46.4% (13/28), *p* = 0.085, respectively). Multivariate logistics analysis showed that high ocular QMG scores at baseline indicated favourable responses to IVMP treatment (OR = 1.781; 95% CI 1.066–2.975; *p* = 0.028). All the adverse events were transient and tolerable.

**Conclusion:**

Our findings suggest that both IVMP and tacrolimus monotherapy hold promise as viable treatment options for OMG patients with unsatisfactory responses to oral prednisone. The study supports the safety and effectiveness of both therapies, with IVMP exhibiting faster improvement and favourable efficacy in patients with high ocular QMG scores.

**Supplementary Information:**

The online version contains supplementary material available at 10.1186/s13023-024-03025-z.

## Introduction

Myasthenia gravis (MG) is an autoimmune disease characterized by the impairment of neuromuscular transmission, mainly due to the loss of acetylcholine receptors (AChRs) and end-plate alterations caused by autoantibodies (Abs) [1]. In 85% of MG patients, pathogenetic Abs mainly target the muscle AChRs, while in some cases, Abs targeting muscle-specific kinase (MuSK) and low-density lipoprotein receptor-related protein 4 (LRP4) can be detected. In about 10% of patients, none of these antibodies is observed, but the clinical presentations and electrophysiological tests enable the diagnosis of seronegative MG [2–4]. On disease onset, most patients show ocular symptoms including ptosis and diplopia, commonly referred to as ocular myasthenia gravis (OMG). The majority of patients will convert to generalized myasthenia gravis (GMG) within a couple of years, if not receiving immunomodulatory therapy [5].

Oral prednisone has been recognized as the first-line therapy for the treatment of OMG. Nevertheless, its long-term use is complicated by numerous adverse effects and some OMG patients reported limited efficacy following the conventional prednisone treatment [5]. New therapies urgently need to be tried. High-dose intravenous methylprednisolone (IVMP) therapy has proved to own advantages in GMG because of rapid improvement and long-term efficacy, allowing a reduction in maintenance prednisolone doses and related adverse effects [6, 7]. In a recent study from Japan, IVMP also turned out to induce faster improvement of ocular symptoms in comparison with low-dose prednisone treatment [8]. However, only 18 patients who had not received any immunotherapy before were enrolled in that study. The finding deserves further confirmation. In addition, non-steroidal immunosuppressive agents, including mycophenolate mofetil (MMF), azathioprine (AZA), methotrexate (MTX), and tacrolimus, are commonly used in conjunction with corticosteroids to reduce the dose and complications of corticosteroids [9]. Recently, the effectiveness of tacrolimus as single-agent immunotherapy in steroid-resistant MG patients has also been demonstrated in several articles [10–13]. This study aimed to evaluate the effectiveness and safety of IVMP and tacrolimus monotherapy in OMG, thus optimizing OMG treatment strategies.

## Materials and methods

### Study design and patients

We performed a retrospective analysis of OMG patients who were treated with IVMP therapy or tacrolimus monotherapy as an alternative choice after receiving unsatisfactory responses to oral prednisone in the Department of Neurology of Xiangya Hospital and Changsha Central Hospital from January 2019 to May 2022. We included patients (≦45 years old) who were diagnosed with OMG for at least 6 months and had not achieved satisfying clinical efficacy after treatment with the conventional prednisone therapy (≥ 0.75 mg/kg/day) for at least 12 weeks prior to enrolment. The unsatisfactory response or the inadequate response was defined as meeting at least one of the following criteria^1,2^: (1) the ocular-quantitative MG (QMG) score improved by < 25%; (2) the prednisone dosage failed to reduce; (3) the MGFA post-intervention state (PIS) didn't improve. Patients who used other immunosuppressants concomitantly with IVMP or tacrolimus and patients who had undergone thymectomy within 48 months were excluded. Finally, 57 patients were included in the study. The diagnosis of OMG was made by experienced neurologists based on the published criteria [5, 14, 15]: (1) fluctuating diplopia, ptosis, ocular motor limitation, or orbicularis oculi weakness at onset without generalization; (2) no prior ocular disease or surgery; (3) at least 2 positive results of the following tests: neostigmine test, serum AChR-Ab or MuSK-Ab, and repetitive nerve stimulation (RNS) test. AChR-Ab titer was detected using AChR-Ab ELISA Kit (RSR, Cardiff, UK), with a concentration of ≥ 0.45 nmol/L defined as positive.

Ocular symptoms were evaluated by the ocular QMG score which includes 3 of the 13 items from the QMG scale (range: 0 to 9 points). Clinical efficacy was defined as a reduction of two or more points on the ocular-QMG scale [13, 16, 17]. We also used the ΔQMG score (the change in ocular QMG scores over time) to evaluate the improvement in OMG symptoms [18]. Clinical data of OMG patients including age, sex, course of disease between onset and initiation of IVMP or oral tacrolimus, presence of thymic abnormality (thymic hyperplasia or thymoma), history of thymectomy, serum Abs status, onset symptoms (ptosis and/or diplopia), presence of autoimmune thyroid disorders and ocular QMG score at baseline were obtained. The follow-up time points were set as 1 month, 3 months, and 6 months after immunotherapy initiation. Adverse events were recorded to monitor the safety of treatment.

### Administration regimen

Patients in the IVMP group (n = 29) were treated with IVMP for 5 to 10 consecutive days, followed by oral corticosteroid tapering. The initial dose of IVMP was 500 mg or 1000 mg per day and then reduced by half every 2–3 days. The methylprednisolone dosing regimen was adjusted by experienced physicians based on each individual’s response to the therapy. A total dose of IVMP of 3000 mg or less, over 3000 to 4250 mg, and over 4250 to 5500 mg was used in the analysis. After IVMP therapy, oral prednisone was given 0.5–1 mg·kg^−1^·d^−1^, and gradually decreased by 5–10 mg/day every 2–4 weeks, up to a minimum dose of 5–10 mg/kg/d. Patients (n = 28) in the tacrolimus group were treated with tacrolimus alone with an initial dose of 1 mg twice a day and then adjusted the dose or added Wuzhi capsule (a traditional Chinese medicine which has been proved to substantially elevate tacrolimus blood concentration) to reach a satisfactory serum concentration (4.8–10 ng/ml) [19] of tacrolimus. The maintenance dose of tacrolimus was 1–3 mg per day according to efficacy and patient tolerance. In order to eliminate the effect of drug interaction on the efficacy assessment, all the patients in the tacrolimus group had stopped taking oral prednisone before the start of tacrolimus treatment.

### Statistical analysis

Statistical analysis was performed with SPSS v26.0. Continuous variables with a non-normal distribution were expressed as the median (interquartile range values (IQRs) p. 25, p. 75), and categorical variables were expressed as counts and percentages. We performed the non-parametric Mann–Whitney *U* test to compare the results of continuous measures and the Chi-square test or Fisher’s exact test to compare categorical outcomes. Point estimates for the pseudo-median difference between groups with 95% CI were calculated using the Hodges–Lehmann method based on the Mann–Whitney U test. Multiple logistic regression analysis was conducted with a variable increasing method using likelihood ratio. Variables with a *p*-value of < 0.10 in bivariate analysis were included in the multivariable model to estimate the odds ratio (OR) and 95% confidence interval (CI). A *p*-value of < 0.05 was considered statistically significant in this study.

## Results

### Baseline characteristics of patients

**Fig. 1:**
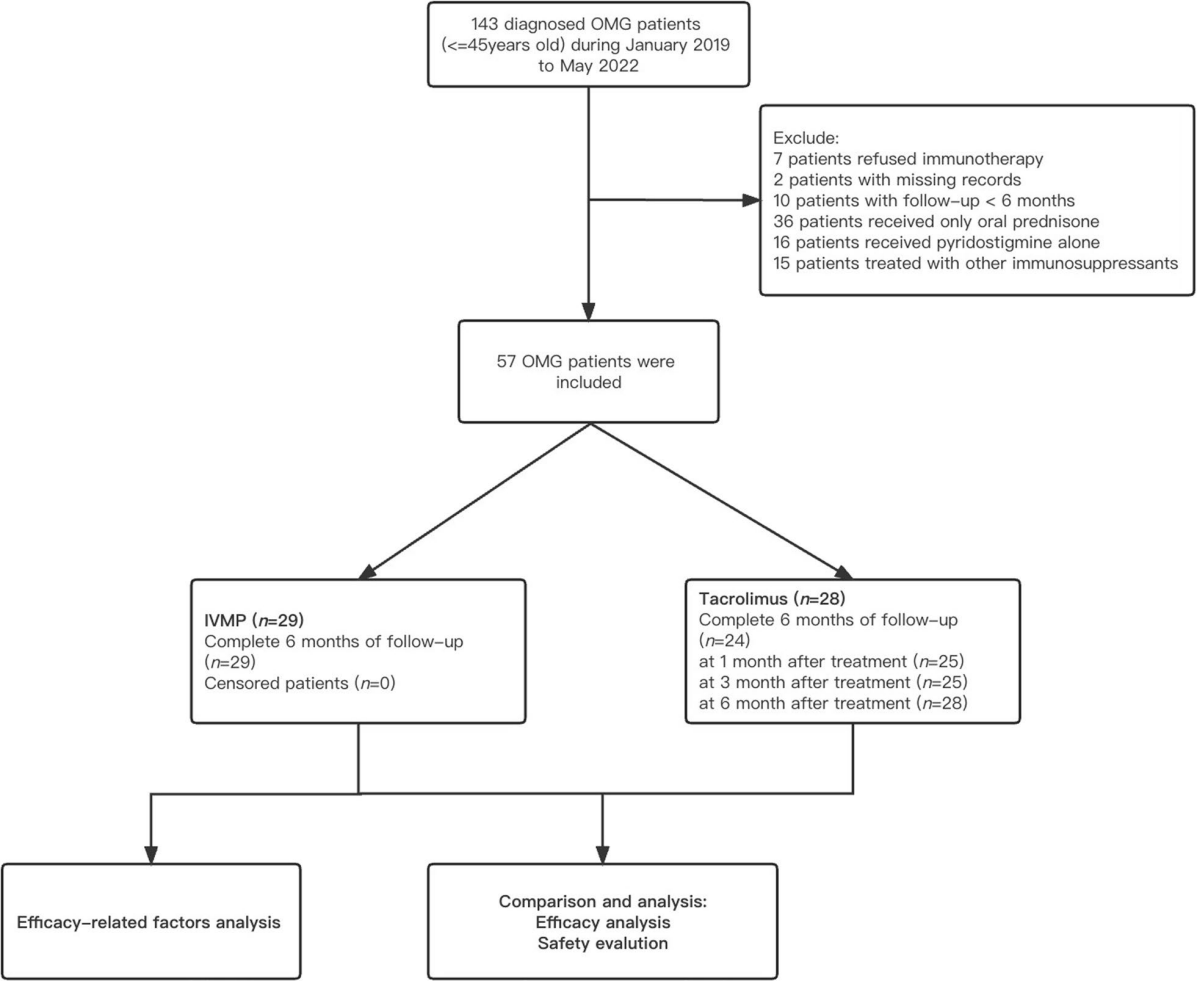
Flowchart of the participants included in this study. OMG, ocular myasthenia gravis; IVMP, intravenous methylprednisolone; *n*, number of patients

The workflow designed for the study was shown in Fig. 1. Of the 143 OMG patients, 57 patients with regular visits at two hospitals met the inclusion criteria and were finally included in this study. Seven patients declined any immunotherapy. Ten patients were eliminated because their follow-up time was less than six months, and two patients were eliminated because their medical records were inadequate with details. We excluded 16 patients receiving pyridostigmine alone, 36 patients receiving only oral prednisone, and 15 patients treated with other immunosuppressants. At last, 57 patients with regular visits at two hospitals met the inclusion criteria and were finally selected.

The baseline characteristics of OMG patients were summarized in Table 1. The enrolled patients had a median age of 20 years (range 11–45 years) and a male-to-female ratio of 1:1.85. The median course of disease was 96 months. According to the MG-related autoantibodies test, 17 cases (29.8%) were seronegative, 39 cases (68.4%) were positive for AChR-Ab, and two cases (3.5%) were positive for MuSK-Ab. No patient tested positive for anti-LRP4-Ab. The median AChR Ab titer was 0.97 nmol/L. Thymectomy was done in six patients (10.5%), and thymoma was detected in four patients with respect to the postoperative pathologic reports. Autoimmune thyroid diseases were present in 22 patients (38.6%), including hyperthyroidism in 21 cases and hypothyroidism in one case, but none of them were in the active stage of thyroid disease. The median ocular QMG score at baseline was 5 points (range 1–9). The neurophysiological data of patients was described in Additional file 1: Table S1.

**Table 1:** Baseline characteristics of IVMP recipients and Tacrolimus recipients

	IVMP	Tacrolimus	*p* value
Age (years) (median [IQR])	20 (16, 24.5)	23.5 (16, 32)	0.230
Male/female (n, %)	14 (48.3%)/15 (51.7%)	6 (21.4%)/22 (78.6%)	0.052
Age at onset (years) (median [IQR])	8 (3.5, 14)	16 (7.3, 26.8)	0.003*
Course of disease (months) (median [IQR])	120 (66, 186)	42 (16, 132)	0.016*
*Antibody status*			
AChR-Ab positivity (n, %)	21 (72.4%)	18 (64.3%)	0.509
MuSK-Ab positivity (n, %)	0	2 (7.1%)	0.237
Seronegative (n, %)	8 (27.6%)	9 (32.1%)	0.707
AChR-Ab titer (nmol/L) (median [IQR])	0.87 (0.52, 3.22)	1.32 (0.40, 3.34)	0.742
*Thymus*			
Thymic hyperplasia (n, %)	2 (6.9%)	4 (14.3%)	0.633
Thymoma (n, %)	2 (6.9%)	2 (7.1%)	1.000
Thymectomy before treatment (n, %)	2 (6.9%)	4 (14.3%)	0.633
Autoimmune thyroid diseases (n, %)	8 (27.6%)	14 (50.0%)	0.082
*Onset symptoms*			
Ptosis (unilateral) (n, %)	18 (62.1%)	17 (60.7%)	0.518
Ptosis (bilateral) (n, %)	11 (37.9%)	9 (32.1%)	
Diplopia (n, %)	13 (44.8%)	14 (50.0%)	0.696
*QMG score at baseline (median [IQR])*			
Ocular QMG score	5 (3, 7.5)	4.5 (3, 5.75)	0.232
QMG score for diplopia	3 (0, 3)	2.5 (0, 3)	0.421
QMG score for ptosis	3 (1, 3)	2.5 (1, 3)	0.513
QMG score for eyelid closure	1 (0, 2)	1 (0, 1)	0.192

IVMP, intravenous methylprednisolone; QMG, quantitative myasthenia gravis; IQR, interquartile range; *p < 0.05

In this study, 29 patients received IVMP, and 28 patients were treated with tacrolimus as single-agent immunotherapy. In the IVMP group, ten patients received 500 mg per day and the others took 1000 mg per day at the beginning of IVMP therapy. The median course of disease in the IVMP group was 120 months, while 42 months in the tacrolimus group (*p* = 0.016). Other clinical features including age, gender, positivity rates of MG-related Abs, AChR-Ab titer, thymus, concomitant diseases, onset symptoms, and ocular QMG score at baseline were not significantly different between the two groups (*p* > 0.05).

### Clinical effectiveness evaluation during the 6-month follow-up

The assessment of ocular QMG scores at 1, 3, and 6 months after treatment did not show significant differences between the two groups (Table 2). At 1 month, the QMG scores for ptosis were lower in the IVMP group than in the tacrolimus group (1 vs. 2,* p* = 0.004). The IVMP group showed faster improvement since the first month after treatment (median ΔQMG score from baseline to 1 month of the IVMP group vs. that of the tacrolimus group: 2 vs. 0, *p* = 0.001; Hodges–Lehmann estimate of the difference in medians, 1; 95% CI, 0–2), while the tacrolimus group started to take effect until 3 months (median ΔQMG scores from baseline to 3 months: 2 vs. 1, *p* = 0. 132; estimated difference, 1; 95% CI, 0–2). Notably, the changes in ocular QMG scores of both groups varied mainly in the previous 3 months, and the change in scores decreased at 6 months (median ΔQMG scores from 3 to 6 months of both groups: 0, *p* = 0.054; estimated difference, 0; 95% CI −1–0), which demonstrated that symptoms tended to stabilize in the long run. From the sub-item of the QMG scale, the IVMP group showed greater improvement in QMG scores for ptosis than the other group (median ΔQMG scores for ptosis from baseline to 1 month: 1 vs. 0, *p* < 0.001; estimated difference, 1; 95% CI, 0–2; median ΔQMG scores for ptosis from baseline to 3 months: 1 vs. 0.5, *p* = 0.018; estimated difference, 1; 95% CI, 0–1).

**Table 2:** Clinical effectiveness evaluation at each follow-up time point

	IVMP (n = 29)	Tacrolimus (n = 28)	Estimated difference (95% CI)^a^	*p* value
*QMG, median (IQR) 1 month after treatment*				
Ocular QMG score	3 (1, 4)	4 (3, 5)	–1 (– 2, 0)	0.078
QMG score for diplopia	1 (0, 3)	1 (0, 3)	0 (0, 0)	0.704
QMG score for ptosis	1 (0, 1.5)	2 (1, 3)	–1 (– 2, 0)	0.004**
QMG score for eyelid closure	0 (0, 1)	1 (0, 1)	0 (0, 0)	0.511
*∆QMG score (baseline—1 month)*				
∆Ocular QMG score	2 (0, 3.5)	0 (0, 1)	1 (0, 2)	0.001**
∆QMG score for diplopia	0 (0, 0)	0 (0, 0)	0 (0, 0)	0.710
∆QMG score for ptosis	1 (0, 2)	0 (0, 0)	1 (0, 2)	< 0.001***
∆QMG score for eyelid closure	0 (0, 1)	0 (0, 0)	0 (0, 1)	0.030*
*3 months after treatment*				
Ocular QMG score	3 (1, 4)	3 (1, 4)	0 (– 1, 1)	0.580
QMG score for diplopia	1 (0, 3)	0 (0, 2.5)	0 (0, 1)	0.339
QMG score for ptosis	1 (0, 1.5)	1 (0, 3)	0 (– 1, 0)	0.100
QMG score for eyelid closure	0 (0, 1)	1 (0, 1)	0 (– 1, 0)	0.210
*∆QMG score (baseline—3 months)*				
∆Ocular QMG score	2 (1, 3.5)	1 (0, 3)	1 (0, 2)	0.132
∆QMG score for diplopia	0 (0, 0.5)	0 (0, 2)	0 (0, 0)	0.575
∆QMG score for ptosis	1 (1, 2)	0.5 (0, 1)	1 (0, 1)	0.018*
∆QMG score for eyelid closure	0 (0, 1)	0 (0, 0)	0 (0, 1)	0.015*
*∆QMG score (1 month—3 months)*				
∆Ocular QMG score	0 (0, 1)	1 (0, 3)	–1 (–1, 0)	0.019*
∆QMG score for diplopia	0 (0, 0)	0 (0, 2)	0 (0, 0)	0.038*
∆QMG score for ptosis	0 (0, 0)	0 (0, 1)	0 (-1, 0)	0.020*
∆QMG score for eyelid closure	0 (0, 0)	0 (0, 0)	0 (0, 0)	0.399
*6 months after treatment*				
Ocular QMG score	3 (1, 4.5)	1.5 (1, 3)	0 (– 1, 2)	0.431
QMG score for diplopia	0 (0, 3)	0 (0, 0.75)	0 (0, 1)	0.086
QMG score for ptosis	1 (0, 2)	1 (0, 2)	0 (– 1, 0)	0.609
QMG score for eyelid closure	0 (0, 1)	0 (0, 1)	0 (0, 0)	1.000
*∆QMG score (baseline—6 months)*				
∆Ocular QMG score	2 (1, 3)	1 (0.25, 4)	0.5 (– 1, 2)	0.432
∆QMG score for diplopia	0 (0, 1.5)	0 (0, 3)	0 (0, 0)	0.496
∆QMG score for ptosis	1 (0.5, 2)	1 (0, 1.75)	0 (0, 1)	0.126
∆QMG score for eyelid closure	0 (0, 1)	0 (0, 1)	0 (0, 1)	0.102
*∆QMG score (3 months—6 months)*				
∆Ocular QMG score	0 (0, 0)	0 (0, 1)	0 (– 1, 0)	0.054
∆QMG score for diplopia	0 (0, 0)	0 (0, 0)	0 (0, 0)	0.477
∆QMG score for ptosis	0 (0, 0)	0 (0, 0)	0 (0, 0)	0.129
∆QMG score for eyelid closure	0 (0, 0)	0 (0, 0)	0 (0, 0)	0.018*

IVMP, intravenous methylprednisolone; QMG, quantitative myasthenia gravis; IQR, interquartile range; CI, confidence interval; *p < 0.05; **p < 0.01; ***p < 0.001

^a^Between-group differences are expressed as a pseudo-median difference calculated with the use of the Hodges–Lehmann estimate based on the Mann–Whitney U test

**Fig. 2:**
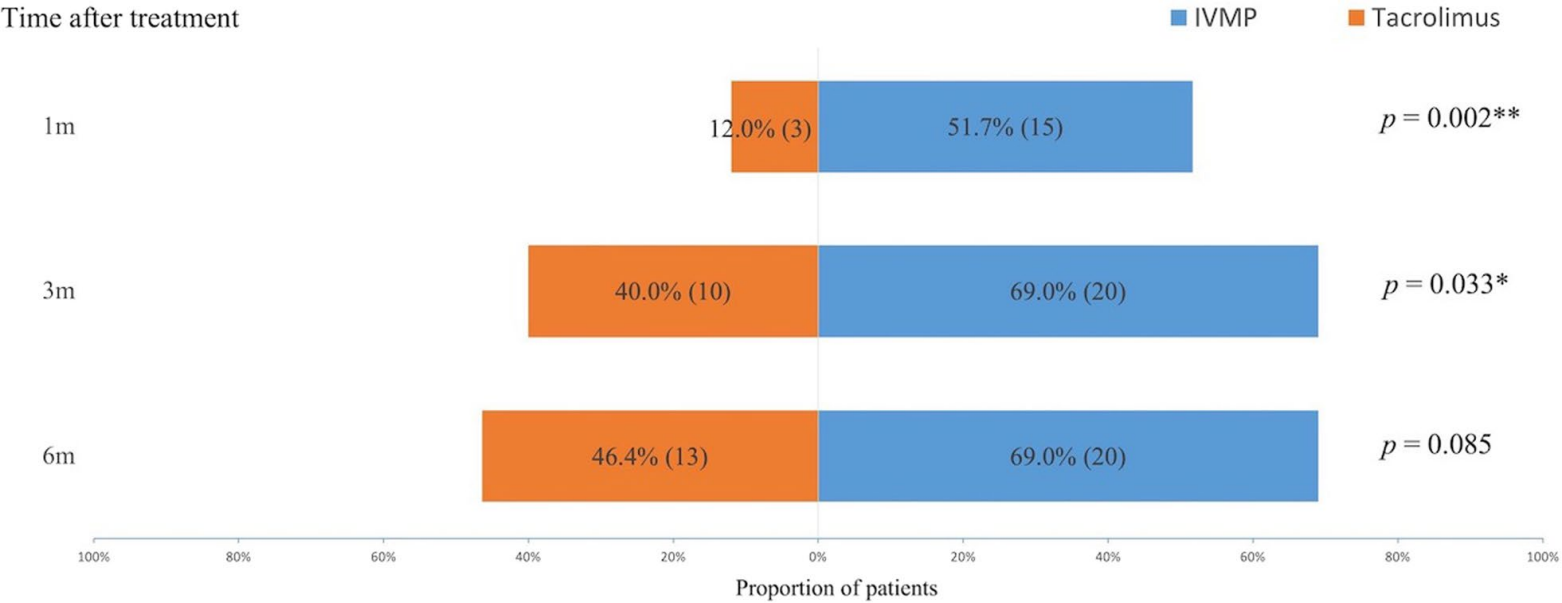
The proportions and numbers of patients achieving clinical efficacy during follow-up. *p < 0.05; **p < 0.01

Clinical effectiveness was evaluated based on quantitative assessment criteria and the results of clinical efficacy at each follow-up time point were displayed in Fig. 2. The proportions of patients reaching the cut-off point for efficacy assessment were obviously higher in the IVMP group than in the tacrolimus group, and were significantly different in the early three months of treatment (1, 3, and 6 months: 51.7% (15/29) vs 12.0% (3/25), *p* = 0.002; 69.0% (20/29) vs 40.0% (10/25), *p* = 0.033; 69.0% (20/29) vs 46.4% (13/28), *p* = 0.085, respectively). During the whole follow-up, there were 20 patients achieving the clinically effective standard in the IVMP group, and 13 patients in the tacrolimus group. Three patients in the tacrolimus group refused the QMG test at 1 and 3 months owing to private reasons. They all reported no progression of the disease and attended to take the test at 6 months after treatment. No patient changed their treatment plan due to initial deterioration or poor treatment effect.

### Factors related to the clinical effectiveness of IVMP

To investigate potential factors associated with the clinical effectiveness of the IVMP therapy, we separated the 29 OMG patients receiving IVMP into two groups according to the change in ocular QMG scores at discharge and compared their clinical characteristics, including age, gender, course of disease, antibody status, thymic abnormalities, thymectomy, autoimmune thyroid diseases, baseline QMG score, period of IVMP therapy, and total dose of methylprednisolone (MP) (Table 3). Subsequently, ten patients were classified into the effective group, and the other 19 patients were classified into the ineffective group. Ocular QMG score and QMG score for ptosis at baseline showed significant differences between the two groups (median ocular QMG score of the effective group vs. that of the ineffective group: 6.50 vs. 4.00, *p* = 0.010; median QMG score for ptosis of the effective group vs. that of the ineffective group: 3.00 vs. 2.00, *p* = 0.013). Then, clinical data, such as gender, concomitant autoimmune thyroid disorders, and baseline QMG scores for ocular items and single ptosis (*p* < 0.1) were included in the multivariate logistic regression model. According to the final analysis, higher ocular QMG scores at baseline (OR = 1.781; 95% CI 1.066–2.975; *p* = 0.028) suggested a better response to the IVMP treatment.

**Table 3:** Potential factors related to clinical effectiveness

Characteristic	Effective group (n = 10)	Ineffective group (n = 19)	*p* value
Male/female (n, %)	2 (20.0%)/8 (80.0%)	12 (63.2%)/7 (36.8%)	0.050
Age (years) (median [IQR])	17.5 (15.5, 24.5)	20 (16, 25)	0.662
Course of disease (months) (median [IQR])	132 (81, 198)	120 (60, 180)	0.836
AChR-Ab positivity (n, %)	7 (70.0%)	14 (73.7%)	1.000
AChR-Ab titer (nmol/L) (median [IQR])	0.67 (0.50, 10.83)	0.90 (0.51, 4.76)	0.850
Onset with ptosis (n, %)			0.114
Unilateral ptosis	4 (40.0%)	14 (73.7%)	
Bilateral ptosis	6 (60.0%)	5 (26.3%)	
Onset with diplopia (n, %)	5 (50.0%)	8 (42.1%)	0.714
Thymic abnormalities (thymoma/hyperplasia), (n, %)	2 (20.0%)	2 (10.5%)	0.592
Thymectomy before treatment (n, %)	0	2 (10.5%)	0.532
Autoimmune thyroid diseases (n, %)	5 (50.0%)	3 (15.8%)	0.083
*QMG score at baseline, (median [IQR])*			
Ocular QMG score	6.5 (5.5, 8)	4 (3, 6)	0.010*
QMG score for diplopia	3 (2.25, 3)	3 (0, 3)	0.198
QMG score for ptosis	3 (3, 3)	2 (1, 3)	0.013*
QMG score for eyelid closure	1.5 (0.75, 2)	1 (0, 2)	0.183
*QMG score at discharge, (median [IQR])*			
Ocular QMG score	3 (1, 4.25)	4 (3, 5)	0.129
QMG score for diplopia	2 (0, 3)	3 (0, 3)	0.661
QMG score for ptosis	1 (0, 1)	1 (1, 3)	0.054
QMG score for eyelid closure	0.5 (0, 1)	1 (0, 2)	0.218
Period of IVMP therapy (days)	6 (5, 7)	7 (6, 9)	0.131
Initial dose of 500 mg/d in IVMP therapy (n, %)	5 (50.0%)	5 (26.3%)	0.244
Total dose of MP in IVMP therapy (n, %)			0.132
≦3000 mg	6 (60.0%)	4 (21.1%)	
3000 mg < , ≦4250 mg	2 (20.0%)	10 (52.6%)	
4250 mg < , ≦5500 mg	2 (20.0%)	5 (26.3%)	

MP, methylprednisolone; IVMP, intravenous methylprednisolone; QMG, quantitative myasthenia gravis; IQR, interquartile range; *p < 0.05

### Safety analysis

During the 6-month follow-up time, no serious adverse reaction was observed in either group. No patient converted to GMG or discontinued treatment. There were a total of 16 side effects reported (5 in the IVMP group, 11 in the tacrolimus group). Metabolic disorders including hyperuricemia and elevated blood glucose mostly occurred in two groups, and hyperlipidaemia was only reported in the tacrolimus group. Gastrointestinal disturbances were the most common side effects in the tacrolimus group, where three patients reported loss of appetite and one patient suffered from nausea. There was one patient in each group who reported insomnia. One patient noticed mild muscle tremors after receiving tacrolimus. A total of three people appeared with initial exacerbation following IVMP treatment. One patient experienced headache and facial pain on the 6th day, and two patients suffered an exacerbation of diplopia on the 4th and 5th days of IVMP treatment. All the side effects were transient and tolerable, and most of them could be alleviated through symptomatic treatment. Adverse reactions during treatment were recorded in Table 4.

**Table 4:** Adverse events during treatment

Adverse events	IVMP	Tacrolimus	p* value*
Pain (n, %)	1 (3.4%)	0	1.000
Insomnia (n, %)	1 (3.4%)	1 (3.6%)	1.000
Tremors (n, %)	0	1 (3.6%)	0.491
Hyperuricemia (n, %)	1 (3.4%)	2 (7.1%)	0.975
Hyperlipidemia (n, %)	0	1 (3.6%)	0.491
Elevated blood glucose (n, %)	2 (6.9%)	2 (7.1%)	1.000
Gastrointestinal symptoms (n, %)	0	4 (14.3%)	0.111

IVMP, intravenous methylprednisolone

## Discussion

In this study, we retrospectively analysed the clinical data of OMG patients who showed unfavourable responses to oral prednisone, thereby receiving IVMP or oral tacrolimus as immunotherapy. By comparing the clinical improvement between the two groups, we found that both therapies could effectively reduce ocular QMG scores and induce remission of ocular symptoms. Moreover, ΔQMG scores from baseline to 1 month were higher in the IVMP group than in the tacrolimus group, while at 3 months, the reverse applied. This result indicated that IVMP had a faster onset on OMG than tacrolimus, which usually took effect after 1–2 months [12, 13]. Other steroid-sparing immunosuppressive drugs, such as AZA, MMF, MTX, or cyclosporine A (CsA), are also used in OMG patients, but all require a several-month course of treatment to show efficacy [20, 21]. Meanwhile, CsA revealed a higher incidence of nephrotoxicity than tacrolimus but exhibits a similar effect [22]. Based on the quantitative evaluation of decreasing QMG scores, both IVMP and tacrolimus monotherapy were effective at achieving clinical efficacy, and more patients showed clinically significant treatment effects in the IVMP group (20, 69.0%) than in the tacrolimus group (13, 46.4%) at the endpoint (*p* = 0.085). The proportion of effective patients differed a lot, especially in the first three months of treatment. Although without long-term follow-up, our study confirmed a favourable short-term efficacy of IVMP in treating OMG.

The feasibility and superiority of tacrolimus as a monotherapy for MG patients has been noted. To date, some studies have documented the efficacy of tacrolimus monotherapy for MG patients. Yagi et al. [10] treated four OMG patients with tacrolimus alone for 24 months and concluded that tacrolimus monotherapy was both safe and effective for the initial treatment of OMG. Fan et al. [11] reported that of the 44 patients (17 OMG and 27 GMG) receiving tacrolimus monotherapy, more than 65% of individuals achieved "MM or better" six months after treatment. This percentage was higher than that in our study, which might be due to the differences in the enrolled patients (with the previous study including both ocular and generalized MG patients), the higher initial daily dose of tacrolimus (2 mg vs. 1 mg), and the evaluation criteria (with the previous study only performing MGADL in the previous six months, which might be highly influenced by patient subjectivity). Previous studies have also found that the efficacy of tacrolimus monotherapy in MG was influenced by many factors, including age, disease severity, AChR-Ab titer, and pharmacogenetics. Duan et al. [12] suggested that MG patients with age < 39, QMG score < 11 points, and AChR-Ab titer < 8.07 nmol/L had a better response to tacrolimus treatment. However, few studies focus on the efficacy-related factors of IVMP in OMG. To eliminate the potential confounding effect of immunosuppressing therapy after IVMP, we focused on the ocular QMG score improvement at hospital discharge when patients just finished IVMP treatment and compared the clinical characteristics of the effective and ineffective groups. We found that there were more females in the effective group (80.0%) at discharge, though males had a similar proportion to females in the IVMP group (male/female: 48.3% vs. 51.7%). In spite of the female predominance of MG patients [23], the effectiveness of IVMP therapy also seems to be better in female patients than in male patients. Additionally, the results of the multivariate logistic analysis showed that patients with higher ocular QMG scores at baseline had better responses to IVMP treatment (OR = 1.781; 95% CI 1.066–2.975; *p* = 0.028). The severity of the disease is inversely correlated with the QMG score [24]. Although the current rating scale has difficulty detecting changes in minor ocular symptoms and the number of patients in this study is limited, our study could partly illustrate that the baseline ocular QMG score appears to be an important predictor of clinical efficacy for OMG patients receiving IVMP treatment.

Initial exacerbation following corticosteroid treatment had been concerning clinicians for decades. A double-blind placebo-controlled study from Sweden showed that IVMP treatment was efficacious and safe in the treatment of moderate MG with no severe side effects observed in participants [7]. Lotan et al. analysed 27 relevant publications regarding the initial deterioration of MG following corticosteroid treatment and found that the rate of MG exacerbation is highest with the administration of cortisone, intermediate with prednisone, and lowest with MP, likewise, indicating the safety and rationality of IVMP therapy [25]. Although the risk of initial exacerbation is usually considered lower in OMG patients compared to GMG patients, studies focused on the safety of IVMP therapy for OMG were insufficient. In our study, IVMP therapy in 29 OMG patients showed a favourable safety profile. Only three cases reported transient adverse reactions following IVMP treatment, which mainly manifested as mild discomfort in the head and facial muscles. Interestingly, there were significantly fewer adverse events recorded in the IVMP group than in the tacrolimus group during 6 months. Tremors and gastrointestinal symptoms were only reported in the tacrolimus group. According to previous studies, age, disease severity, generalized MG, presence of thymoma, and thymectomy before IVMP are important factors related to initial exacerbation [25–27]. Bae et al. [26] first identified that the age of patients in the exacerbated group was significantly higher than that of patients in the non-exacerbated group (52.3 ± 13.4 vs. 41.1 ± 15.4) in a retrospective study with 55 MG patients. Considering the patient’s overall physical condition and the potential risk of high-dose methylprednisolone therapy, we didn’t include patients older than 45 years in this study. Our results should be carefully applied in clinical practice, especially for middle-aged and elderly MG patients. Further studies with larger populations are needed.

Almost half a century after the first report of MP used for the treatment of MG [28], there is still no consensus on treatment protocol or guidelines of MP in clinical practice, especially for OMG patients. Protocols of IVMP treatment for MG are remarkably different. In one study, 1000 mg per day of MP was repeatedly administered monthly [8]; in another study, 3 days of 250 mg, 500 mg, or 1000 mg IVMP was administered weekly [27]. MP was intravenously at a dose of 1–2 g/day in most studies, without dose de-escalation [6, 7, 29, 30]. In our study, the initial dose of MP was 500 mg or 1000 mg per day and was then reduced by half every 2–3 days. Subtle differences existed in the dosage of MP among patients, but there was no significant difference in therapeutic efficacy between patients with different dosages of MP (initial dose, 500 mg vs. 1000 mg, *p* = 0.244). We supposed that one primary reason could be related to the initial disease severity at presentation. Higher initial doses of MP were more likely to be used when the symptoms were more pronounced and aggressive. Another critical aspect to consider is that the response to immunotherapies like IVMP could vary significantly between individuals. In addition, individual differences in the metabolism of MP could influence therapeutic outcomes. Patients who metabolize the drug faster might require higher doses for optimal therapeutic effects. Guo et al. [31] found that visual improvements in neuromyelitis optica (NMO) patients following IVMP treatment did not significantly differ between patients treated with doses of 500 mg/day and 1000 mg/day, which is similar to our result. More importantly, these data suggested that using a lower methylprednisolone dose could achieve the same therapeutic effect as a higher one, which showed great clinical significance because it would reduce the occurrence of adverse events with methylprednisolone and relieve the financial burdens of patients by reducing the doses of MP. Although the result still needs to be confirmed in prospective randomized clinical trials, our study provides preliminary evidence for the future selection of MP dosage for OMG patients.

In clinical practice, the decision to opt for an ocular MG treatment strategy is multifactorial and often individualized based on the clinician's judgment and the patient's specific circumstances. Factors such as disease severity, the presence of contraindications, previous therapeutic responses, and patient preferences indeed shape therapeutic decisions. Although there are concerns about the early exacerbation caused by IVMP, serious adverse events are rare in OMG treatment. From the patient's perspective, compared with the high cost of tacrolimus and the severe side effects caused by long-term and much use of oral prednisone, IVMP is a relatively economical and proper choice for OMG patients. However, currently in China, patients still require short-term hospitalization in order to receive IVMP therapy due to the intravenous administration method and safety concerns, whereas through outpatient visits, patients can simply and consistently receive tacrolimus treatment.

There are several limitations in our study. The major one is its retrospective and non-randomized nature. The precise information about the dose and cumulative dose of oral prednisone before treatment was incomplete, which might result in an imbalance between groups at baseline. Second, the study has a relatively small sample number and therefore doesn't meet the requirement of events per variable in logistic regression, which may influence the stability of statistical results. Patients with inadequate medical records or interrupted follow-up were excluded from the analysis because we could not obtain precise ocular-QMG scores and other important therapy information. In addition, middle-aged and elderly patients (> 45 years old) and patients with anti-LRP4-Ab positive were not included and there were significant differences in the disease duration and onset-age of patients between the two groups. Thus, potential selection bias may exist in this study. Finally, large prospective clinical trials over a longer follow-up period are needed to evaluate the efficacy of IVMP and tacrolimus monotherapy in OMG patients and verify the results of this study.

## Conclusion

In conclusion, our study suggests that both IVMP and tacrolimus monotherapy have great potential to be used to treat OMG patients with unsatisfactory responses to oral prednisone. IVMP therapy induces a faster-acting in comparison and shows favourable efficacy in OMG patients, especially with high ocular QMG scores.

### Supplementary Information

**Additional file 1:** RNS test results of the patients.

## Data Availability

All data generated or analysed during this study are included in this published article, and its supplementary information is available in Figshare with the identifier https://doi.org/10.6084/m9.figshare.24226114.

## Abbreviations

IVMP: Intravenous methylprednisolone (IVMP)
OMG: Ocular myasthenia gravis (OMG)
QMG: Quantitative myasthenia gravis (QMG)
AChRs: Acetylcholine receptors (AChRs)
Abs: Autoantibodies (Abs)
MuSK: Muscle-specific kinase (MuSK)
LRP4: Low-density lipoprotein receptor-related protein 4 (LRP4)
GMG: Generalized myasthenia gravis (GMG)
MMF: Mycophenolate mofetil (MMF)
AZA: Azathioprine (AZA)
MTX: Methotrexate (MTX)
RNS: Repetitive nerve stimulation (RNS)
IQRs: Interquartile range values (IQRs)
OR: Odds ratio (OR)
CI: Confidence interval (CI)
MP: Methylprednisolone (MP)
CsA: Cyclosporine A (CsA)
