# Supplementary material for: Comparative effectiveness and safety of intravenous methylprednisolone and tacrolimus monotherapy in ocular myasthenia gravis with unsatisfactory prednisone responses: a retrospective study
Source: Orphanet J Rare Dis. 2024 Jan 19;19:19. doi: 10.1186/s13023-024-03025-z (PMC10799357; doi:10.1186/s13023-024-03025-z)
Supplement: Supplementary file 1 — Additional file 1: RNS test results of the patients. [file 13023_2024_3025_MOESM1_ESM.docx]

Supplementary Table S1. RNS test results of the patients

| RNS test positive | IVMP (n=18) | Tacrolimus (n=15) | *p* value |
| --- | --- | --- | --- |
| Facial nerve (n, %) | 7 (38.9%) | 5 (33.3%) | 1.000 |
| Ulnar nerve (n, %) | 3 (16.7%) | 1 (6.7%) | 0.607 |
| Accessory nerve (n, %) | 0 | 1 (6.7%) | 0.455 |
| Axillary nerve (n, %) | 2 (11.1%) | 2 (13.3%) | 1.000 |
